# The Pro-Tumorigenic Role of Chemotherapy-Induced Extracellular HSP70 from Breast Cancer Cells via Intratumoral Macrophages

**DOI:** 10.3390/cancers15061903

**Published:** 2023-03-22

**Authors:** Mio Yamaguchi-Tanaka, Kiyoshi Takagi, Yasuhiro Miki, Ai Sato, Erina Iwabuchi, Minoru Miyashita, Takashi Suzuki

**Affiliations:** 1 Department of Pathology and Histotechnology, Graduate School of Medicine, Tohoku University, Sendai 980-8575, Japan; 2 Department of Nursing, Faculty of Medical Science & Welfare, Tohoku Bunka Gakuen University, Sendai 981-8551, Japan; 3 Department of Breast and Endocrine Surgical Oncology, Graduate School of Medicine, Tohoku University, Sendai 980-8575, Japan; 4 Department of Anatomic Pathology, Graduate School of Medicine, Tohoku University, Sendai 980-8575, Japan; 5 Department of Pathology, Tohoku University Hospital, Sendai 980-8574, Japan

**Keywords:** breast cancer, macrophage, chemotherapy, tumor microenvironment, HSP70, TGF-β

## Abstract

**Simple Summary:**

Resistance to chemotherapy is an important problem to be solved in breast cancer research. Tumor-associated macrophages (TAMs) contribute to breast cancer progression, including chemoresistance, and it is important to clarify the altered functions of macrophages following chemotherapy to improve prognosis of breast cancer patients. Here, we conducted in vitro experiments and immunohistochemistry in 116 breast carcinoma tissues to determine whether the secretion of heat shock protein (HSP) 70 from breast cancer cells following chemotherapy affects macrophage function. It was revealed that extracellular HSP70 levels increased following chemotherapy and enhanced the pro-tumorigenic effects of TAMs either directly or indirectly by regulating the expression of transforming growth factor (TGF)-β in breast cancer cells. Immunohistochemistry demonstrated that HSP70 functions as a poor prognostic factor in conjunction with macrophage infiltration. Targeting HSP70 may therefore be useful in regulating the tumor microenvironment in breast cancer tissues and improving the prognosis of breast cancer patients following chemotherapy.

**Abstract:**

Tumor-associated macrophages (TAMs) contribute to tumor progression and chemoresistance; it is therefore important to clarify the altered functions of macrophages following chemotherapy. While extracellular heat shock protein (HSP) 70 is associated with therapeutic resistance, the effects of HSP70 on TAMs remain largely unknown. Here, we conducted in vitro experiments and immunohistochemistry in 116 breast carcinoma specimens to determine whether the secretion of HSP70 from breast cancer cells following chemotherapy affects macrophage function. It was revealed that the interaction of epirubicin (EPI)-exposed breast cancer cells with macrophages enhanced tumor progression, and EPI promoted the secretion of extracellular HSP70 from breast cancer cells. The expression of pro-tumorigenic macrophage marker CD163 was decreased in macrophages treated with a conditioned medium (CM) from HSP70-silenced breast cancer cells. Breast cancer cells treated with CM from HSP70-silenced breast cancer cells showed decreased expression of transforming growth factor (TGF)-β, and the pro-tumorigenic effects of macrophages were impaired when TGF-β signaling was inhibited. Immunohistochemistry demonstrated that HSP70 served as a poor prognostic factor in conjunction with macrophage infiltration. It was therefore concluded that extracellular HSP70 levels increased following chemotherapy and enhanced the pro-tumorigenic effects of TAMs, either directly or indirectly, by regulating TGF-β expression in breast cancer cells.

## 1. Introduction

Cytotoxic chemotherapy is widely used for aggressive types of breast cancer and improves the prognosis for breast cancer patients. In addition, chemotherapy is the mainstay of treatment for triple-negative breast cancers (TNBCs), which do not express the estrogen receptor (ER), progesterone receptor (PR), or human epidermal growth factor receptor 2 (HER2). However, about 25% of breast cancer patients experience distant metastasis after adjuvant chemotherapy [1]; therefore, the molecular mechanism of resistance to chemotherapy needs to be elucidated to further improve the clinical outcomes of patients.

The tumor microenvironment (TME) includes tumor cells and stromal cells such as macrophages, leukocytes, and fibroblasts, and interactions between these cells play an important role in tumor progression [2]. In particular, tumor-associated macrophages (TAMs) represent a significant component of the TME. Macrophages are subdivided into the M1 and M2 phenotypes, which have different functions and cell surface markers [3]. M2 macrophages, which express CD163 [4], are the predominant phenotype of TAMs and contribute significantly to tumor malignancy by promoting cell proliferation, invasion, angiogenesis, immunosuppression, and metastasis [5,6,7,8,9,10,11]. Meanwhile, M1 macrophages produce pro-inflammatory cytokines and mediate the antitumor immune response.

Increased infiltration of macrophages in breast cancer tissues is frequently observed following chemotherapy [12]. It is involved in the development of resistance to breast cancer chemotherapy and correlates with poor clinical outcomes [13,14,15]. A previous study showed that targeting these macrophages by inhibiting either the myeloid cell receptors colony-stimulating factor-1 receptor (CSF1R) or C-C motif chemokine receptor (CCR) 2 improved chemotherapeutic efficacy, inhibited metastasis, and promoted antitumor T-cell responses [16]. However, the systemic depletion of macrophages may disrupt innate immunity and aggravate the adverse effects in patients who have undergone chemotherapy. Therefore, it is important to understand cancer cell–macrophage communication to specifically target TAMs and improve responses to chemotherapy.

Heat shock protein (HSP) 70 is known as a stress-inducible chaperone that facilitates the correct folding of nascent and damaged misfolded proteins [17]. Various types of malignant cells, including breast cancer cells, express high amounts of HSP70 [18]. Importantly, HSP70 has also been demonstrated to be released into extracellular space under stressful stimuli, including infection or tissue damage; it also modulates the innate immune system by inducing the secretion of pro-inflammatory cytokines from antigen-presenting cells [19,20]. On the other hand, HSP70 has recently been reported to enhance the production of anti-inflammatory cytokines from cells with immunosuppressive effects, such as regulatory T cells [21,22] and myeloid-derived suppressor cells [23,24], activating their immunosuppressive functions. Although the role of extracellular HSP70 in the tumor microenvironment is not yet fully understood, it has been reported to activate neutrophils through Toll-like receptor (TLR) 2/4 and to cause the production of reactive oxygen species and the release of IL-8, promoting tumor angiogenesis and metastasis [25,26,27]. It has also been reported that extracellular HSP70 enhances tumor growth and resistance to chemotherapy by activating MDSC by binding to TLR2 in various cancers [23,28].

HSP70 lacks a specific signal peptide that targets proteins for secretion [29]. Therefore, extracellular HSP70 is not secreted by the endoplasmic reticulum (ER)–Golgi classical secretory pathway but by different mechanisms such as secretory lysosomes [30], oligomerization, binding to phosphatidylserine [31,32,33,34], and penetration through the lipid bilayer and the structure of lipid rafts [35,36]. Furthermore, HSP70 is transported by small extracellular vesicles (sEVs), including exosomes, which are less than 200 nm in diameter [37] and are either expressed on the membrane of sEVs or contained inside sEVs. Based on the mechanisms of biogenesis and size, two classes of EVs have been identified [38]. Ectosomes, including microvesicles and oncosomes, are formed by direct cell membrane budding (100–1000 nm), and exosomes are nanosized vesicles (30–150 nm) produced via the endocytic pathway. EVs contain various proteins, lipids, and nucleic acids, and EV-mediated intercellular delivery plays an important role in inter-cell communication within the TME [39,40,41,42,43]. However, the effects of extracellular HSP70 on TAMs remain largely unknown in breast cancers.

Therefore, we focused on the possible interaction between TAMs and breast cancer cells via extracellular HSP70 following chemotherapy. We hypothesized that the altered secretion of extracellular HSP70 partially contained in the sEVs secreted from breast cancer cells following chemotherapy affects the pro-tumorigenic effects of macrophages, either directly, or indirectly by regulating the expression profile of the cytokines in breast cancer cells.

## 2. Materials and Methods

### 2.1. Cell Lines and Chemicals

The human TNBC cell lines MDA-MB-231 and MDA-MB-453 and the human leukemic cell line THP-1 were obtained from the American Type Culture Collection (Manassas, VA, USA) and the Japanese Collection of Research Bioresources Cell Bank (JCRB; Osaka, Japan), respectively. Breast cancer cells were cultured in RPMI-1640 (Fujifilm Wako, Osaka, Japan) with 10% fetal bovine serum (FBS) (Biosera, Nuaillé, France). THP-1 cells were cultured in RPMI-1640 with 10% FBS and 0.1 mM 2-mercaptoethanol (Fujifilm Wako). All cells were incubated at 37 °C under 5% CO_2_. We performed routine checks of cell cultures for mycoplasma contamination.

### 2.2. Differentiation of THP-1 to Macrophage

To differentiate the THP-1 cells into macrophages, they were stimulated by 20 nM phorbol 12-myristate 13-acetate (PMA; Fujifilm Wako) for 72 h, as reported previously [44]. Differentiation was confirmed by the adherence of the cells to the bottom of dishes or culture plates. Galunisertib was purchased from MedChemExpress (Middlesex, NJ, USA).

### 2.3. Coculture Experiment

MDA-MB-231 and MDA-MB-453 cells were cocultured with THP-1-derived macrophages using ThinCerts™ (pore size 0.4 μm, Greiner bio-one, Monroe, NC, USA). The THP-1 cells differentiated into macrophages and were cocultured for 72 h with MDA-MB-231 cells or MDA-MB-453 cells, which had been treated with or without epirubicin (EPI; 0.1 and 0.5 μM, Fujifilm Wako) for 6 h before coculture and washed with PBS 3 times. The THP-1 cells were further cultured for 72 h in a fresh medium without breast cancer cells; finally, the conditioned medium (CM) (THP-1/BC-EPI CM) was collected.

### 2.4. Cell Proliferation Assay, Chemoresistance Assay, and Wound Healing Assay

The MDA-MB-231 and MDA-MB-453 cells were seeded in a 96-well plate (cell proliferation assay; 7500 cells/well, chemoresistance assay; 10,000 cells/well) and allowed to attach for 24 h. CM from THP-1-derived macrophages (50% *v*/*v*) was added, and the cell viability was measured using a Cell Counting Kit-8 (Dojindo Molecular Technologies, Kumamoto, Japan) for 3–4 days. Absorbance at 450 nm was determined using a Bio-Rad iMark plate reader (Bio-Rad Laboratories Inc., Hercules, CA, USA). For the chemoresistance assay, MDA-MB-231 and MDA-MB-453 cells were treated with CM from macrophages along with 0.1 μM EPI.

The cell migration ability of the breast cancer cells was evaluated by a wound healing assay using culture inserts (Platypus Technologies, Madison, WI, USA). The MDA-MB-231 and MDA-MB-453 cells were seeded in a 96-well plate with culture inserts at >90% confluency. The culture inserts were removed, and then CM from macrophages (50% *v*/*v*) was added. The remaining gaps were evaluated using the Image J 1.52a software (https://imagej.nih.gov/ij/, accessed on 1 February 2023). The relative migration rate was evaluated as the ratio (%) to those at the removal of the culture inserts (0 h).

### 2.5. Collection of the Conditioned Medium and Downstream Experiments

The CM was collected and used for the stimulation of other cells or for Western blotting. CM was centrifuged at 1500 rpm for 3 min to remove cell debris, and the supernatant was used in the subsequent experiments. For Western blotting, MDA-MB-231 and MDA-MB-453 cells were cultured in a serum-free RPMI-1640 medium 24 h before CM collection so as not to carry over the proteins from the FBS. For sEV isolation, these cells were cultured using exosome-depleted FBS (System Biosciences, Palo Alto, CA, USA). For the neutralization of HSP70, the CM was preincubated with the HSP70 antibody (Table S1) or a mouse IgG1 isotype control (5 μg/mL, R&D Systems Inc., Minneapolis, MN, USA) for 1 h at 37 °C, and the THP-1-derived macrophages and MDA-MB-231 cells were then treated with the CM.

### 2.6. Isolation of sEVs

The ExoQuick Exosome Precipitation Solution (System Biosciences), which has been widely used for the isolation of exosomes in previous studies [45,46,47,48], was used to isolate the sEVs according to manufacturer’s protocol. Briefly, MDA-MB-231 and MDA-MB-453 cells were cultured in RPMI-1640 with 10% exosome-depleted FBS for 24 h and treated with or without EPI (0.5 and 1 μM) and docetaxel (DTX; 1 and 5 nM, Fujifilm Wako) for 4 days. The CM was collected and thoroughly mixed with 0.2 volumes of ExoQuick solution, then incubated at 4 °C overnight. The sample was centrifuged at 1500× *g* for 30 min, and the supernatant was removed and further centrifuged at 1500× *g* for 5 min. The supernatant was completely removed, and the sEVs pellet was suspended in an SDS sample buffer (125 mM Tris-HCl (pH 6.8), 4% SDS, 10% glycerol, and 0.01% bromophenol blue).

### 2.7. Western Blotting

Western blotting was performed as per the methodology described in previous reports [49,50,51]. The cells were lysed using an M-PER Mammalian Protein Extraction Reagent (Pierce Biotechnology, Rockford, IL, USA) containing a Halt Protease Inhibitor Cocktail (Sigma Aldrich, St. Louis, MO, USA). CM was mixed with four volumes of cold acetone and incubated at −20 °C for 1 h, followed by centrifugation at 10,000× *g* for 20 min. The pellet was washed with ethanol and resuspended in the SDS sample buffer, and the protein extracts (10 μg) were separated using SDS-PAGE (10% acrylamide gel). Information about the primary antibodies is listed in Table S1. The HRP-conjugated secondary antibody was purchased from GE Healthcare (Buckinghamshire, UK) and the immunoreactive bands on the membrane were visualized using ImmunoStar Reagents (Fujifilm Wako) and the LAS-4000 image analyzer (Fuji Photo Film Co., Tokyo, Japan). β-actin and CD63 were used as loading controls for total cell lysate and sEVs lysate, respectively. Densitometric analysis was performed using ImageJ 1.52a software. The protein level of HSP70 and TGF-β was normalized by that of the loading controls and the data are presented as the fold change. The uncropped blots and molecular weight markers are shown in Supplemental Materials.

### 2.8. Fluorescence Immunocytochemistry

MDA-MB-231 and MDA-MB-453 cells were treated with EPI (10 μM) for 30, 60, and 180 min and fixed with 4% paraformaldehyde phosphate-buffered solution (Fujifilm Wako), followed by permeabilization with 0.1% Triton X-100 (Fujifilm Wako) in PBS. After blocking with 10% FBS in PBS, the cells were incubated overnight with HSP70 primary antibody (Table S1) at 4 °C. The cells were incubated with Alexa Fluor 594-AffiniPure anti-mouse IgG (1:100, cat. no. 115-585-003, Jackson Immuno Research, West Grove, PA, USA) and 0.5 μg/mL DAPI (Fujifilm Wako) for the counterstaining of nuclei. The fluorescence was visualized using an Olympus FSX100 microscope (Olympus, Tokyo, Japan). The fluorescence intensity of HSP70 was analyzed using Image J 1.52a software. 

### 2.9. Small Interfering RNA Transfection

Two siRNAs for HSP70 (siHSP70 #1 and siHSP70 #2, Table S2) were purchased from Ajinomoto Bio-Pharma Services, Inc. (Osaka, Japan). The MISSION siRNA Universal Negative Control (Sigma Aldrich) was used as the negative control (siCTRL). The siRNAs were transfected with MDA-MB-231 (20 nM) and MDA-MB-453 cells (50 nM) using the Lipofectamine RNAi MAX transfection reagent (Thermo Fisher Scientific, Waltham, MA, USA). For the collection of the CM to treat THP-1-derived macrophages or breast cancer cells, the siR-NAs were transfected with MDA-MB-231 and MDA-MB-453 cells, and the medium was replaced with a fresh medium 24 h after transfection to remove the remaining siRNAs. These cells were further cultured for 72 h and, finally, the CM (BC-siHSP70 CM) was collected.

### 2.10. Real-Time PCR

RNA extraction was carried out using the TRI Regent (Molecular Research Center, Inc., Cincinnati, OH, USA), and cDNA was synthesized using a ReverTra Ace qPCR RT Master Mix with a gDNA Remover (TOYOBO CO. LTD., Osaka, Japan). Real-time PCR was performed using a THUNDERBIRD SYBR qPCR Mix (TOYOBO) and a LightCycler nanosystem (Roche Diagnostics Japan, Tokyo, Japan). The sequences for the PCR primer sets are listed in Table S2. The mRNA expression levels of *CD163*, *TGFB1*, *IL10*, and *MMP2* were normalized by *RPL13A* and presented as fold changes (mean ± S.D., *n* = 3) compared to the negative control.

### 2.11. Patients and Tissues

In total, 116 specimens of invasive breast carcinoma tissues were obtained from female patients who had undergone surgical treatment from 2007 to 2008 at Tohoku University Hospital. All specimens had been fixed with formalin and embedded in paraffin wax. Of these patients, 59 had received adjuvant chemotherapy. The details of the chemotherapy regimens are as follows: anthracycline-based therapy, 21 patients; taxane-based therapy, 15 patients; anthracycline- + taxane-based therapy, 12 patients; others, 11 patients (CMF (cyclophosphamide + methotrexate + 5-fluorouracil), 6 patients; UFT (tegafur-uracil), 1 patient; furtulon, 1 patient; unknown, 3 patients). Disease-free survival was defined as the period from the date of surgery to that of the first locoregional recurrence or distant metastasis within the follow-up time, and the median time was 59 months. Breast-cancer-specific survival was defined as the period from surgery to death from breast cancer, and the median follow-up time was 61 months. The research protocol was approved by the Ethics Committee at the Tohoku University Graduate School of Medicine (approval number 2021-1-503).

### 2.12. Immunohistochemistry

Table S1 shows information related to antibodies. Immunohistochemistry for HSP70 was performed using a Histofine kit (Nichirei Bio, Inc., Tokyo, Japan). The antigen–antibody reaction was visualized using 3,3′-diaminobenzidine solution, and hematoxylin was used for counterstaining. The immunohistochemical statuses of CD163, ER, PR, HER2, and Ki67 were available from previous studies [52,53].

### 2.13. Scoring of HSP70 Immunoreactivity

HSP70 immunoreactivity was detected in both the cytoplasm and nucleus of breast carcinoma cells, and we focused on the cytoplasmic HSP70 staining of breast carcinoma cells, which was considered to reflect extracellular HSP70 activity. Cytoplasmic HSP70 immunoreactivity was considered positive when the cases had more than 10% positive carcinoma cells.

### 2.14. Statistical Analyses

Statistical analyses were performed using the JMP Pro 15.0.0 software (SAS Institute, Cary, NC, USA). The χ^2^ test or Mann–Whitney U test was used to evaluate the correlation between HSP70 expression and clinicopathological parameters. Disease-free and breast-cancer-specific survival curves were generated according to the Kaplan–Meier method, and statistical significance was examined by a log-rank test. The proportional hazard model (COX) was used for the univariate and multivariate analyses. Scheffe’s F test was used in the in vitro experiments. The data are presented as the mean ± S.D (*n* = 3) and *p* < 0.05 was considered significant in this study.

## 3. Results

### 3.1. Altered Characteristics of Macrophages following Chemotherapy in Breast Cancer

**Figure 1:**
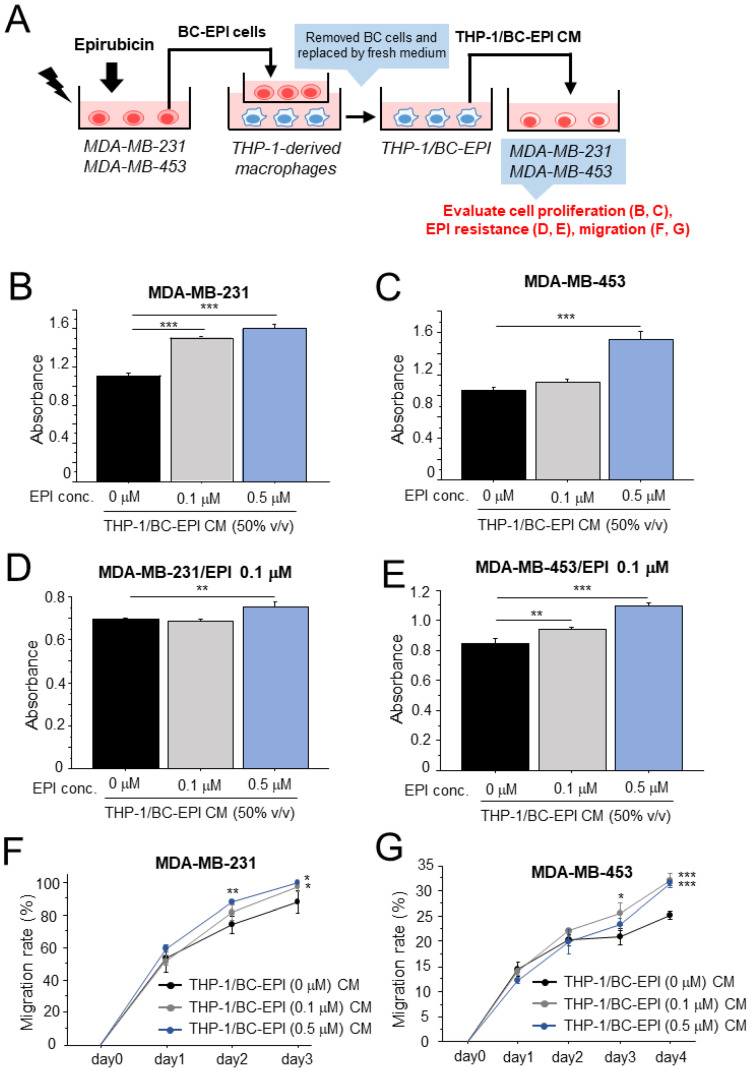
Effects of breast cancer cells following chemotherapy on the pro-tumorigenic ability of macrophages. (**A**): Schematic image of coculture experiments to investigate the effects of breast cancer cells treated with epirubicin (EPI) on the pro-tumorigenic activity of macrophages. (**B**–**G**): Cell proliferation (**B**,**C**), resistance to EPI (**D**,**E**), and migration (**F**,**G**) assays in MDA-MB-231 (**B**,**D**,**F**) and MDA-MB-453 (**C**,**E**,**G**) cells using conditioned medium (CM) from macrophages that had interacted with EPI-treated breast cancer cells (THP-1/BC-EPI CM, 50% *v*/*v*). The concentration of EPI is indicated in the figure. Cell viability was measured 72 h after the CM treatment. Cell migration was examined with a wound healing assay using MDA-MB-231 and MDA-MB-453 cells treated with THP-1/BC-EPI CM (50% *v*/*v*). The data are presented as the mean ± S.D. (*n* = 3). * *p* < 0.05, ** *p* < 0.01, *** *p* < 0.001 vs. control (EPI 0 μM). BC: breast cancer, CM: conditioned medium, EPI: epirubicin.

We first examined our hypothesis using the human leukemic cell line THP-1 and human TNBC cell lines MDA-MB-231 and MDA-MB-453 because cytotoxic chemotherapy is mainly used for aggressive types of breast cancer, including TNBC. THP-1-derived macrophages were cocultured with MDA-MB-231 and MDA-MB-453 cells that had been treated with EPI, and they were further cultured in a fresh medium without breast cancer cells (Figure 1A). The CM from these macrophages (THP-1/BC-EPI CM) significantly promoted cell proliferation (Figure 1B,C), survival in the presence of EPI (Figure 1D,E), and the migration (Figure 1F,G) of MDA-MB-231 and MDA-MB-453 cells compared with the CM from macrophages that interacted with naïve breast cancer cells. The interaction of macrophages with naïve breast cancer cells did not significantly upregulate the pro-tumorigenic effects of macrophages (Figure S1A–F). To nullify the possibility of EPI carryover to macrophages from breast cancer cells, we confirmed that low doses of EPI did not directly affect the M2 polarization of THP-1-derived macrophages (Figure S2A), and CM from these macrophages (THP-1-EPI CM) did not promote the cell proliferation of breast cancer cells (Figure S2B).

### 3.2. Effects of Chemotherapy on Extracellular HSP70 Secretion in Breast Cancer Cells

**Figure 2:**
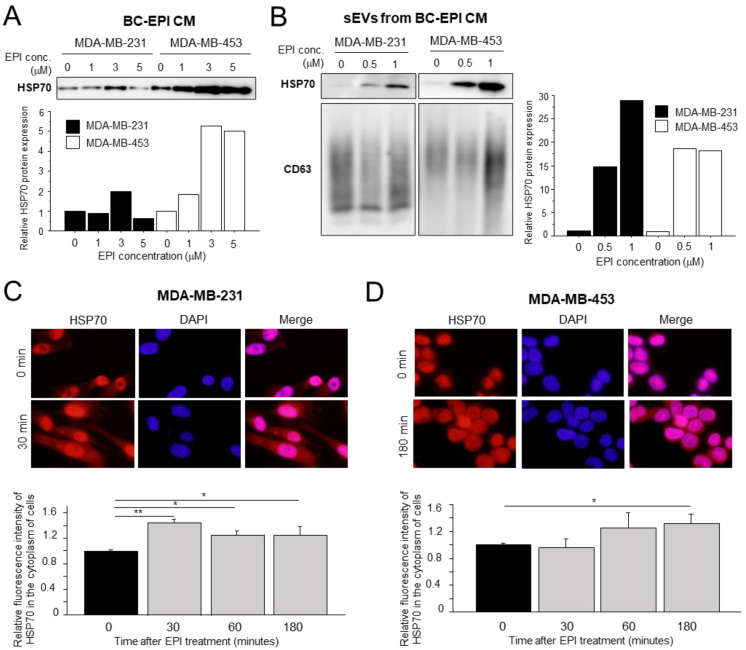
Expression of heat shock protein (HSP) 70 in breast cancer cells after chemotherapy. (**A**,**B**): HSP70 protein level in the CM (**A**) and small extracellular vesicles (sEVs) (**B**) from MDA-MB-231 and MDA-MB-453 cells treated with EPI for 24 h (CM) or 96 h (sEVs). CD63 was used as the sEV marker. (**C**,**D**): Fluorescence immunocytochemistry for HSP70 (red) in MDA-MB-231 (**C**) and MDA-MB-453 (**D**) cells treated with EPI (10 μM). The cytoplasmic HSP70 fluorescence intensity of 40 cells per well was measured using ImageJ software at 40× objective magnification. The average scores were calculated and presented as the fold change (mean ± S.D., *n* = 3). * *p* < 0.05, ** *p* < 0.01 vs. control (0 min). BC: breast cancer, CM: conditioned medium, EPI: epirubicin, sEVs: extracellular vesicles. The uncropped blots are shown in Supplementary Materials.

We next investigated the secreted factors induced by chemotherapy in breast cancer cells and found that HSP70 protein was increased in the CM from breast cancer cells treated with EPI (BC-EPI CM) (Figure 2A). Notably, HSP70 was also enriched in the sEVs secreted into BC-EPI CM in a dose-dependent fashion (Figure 2B), and the same results were obtained using DTX (Figure S3A). Total HSP70 protein expression was not altered in breast cancer cells treated with EPI (Figure S3B). Furthermore, using fluorescent immunocytochemistry, we found that the HSP70 protein was significantly translocated from the nucleus into the cytoplasm of breast cancer cells by EPI (Figure 2C,D).

### 3.3. Direct or Indirect Effects of Extracellular HSP70 on Macrophages

**Figure 3:**
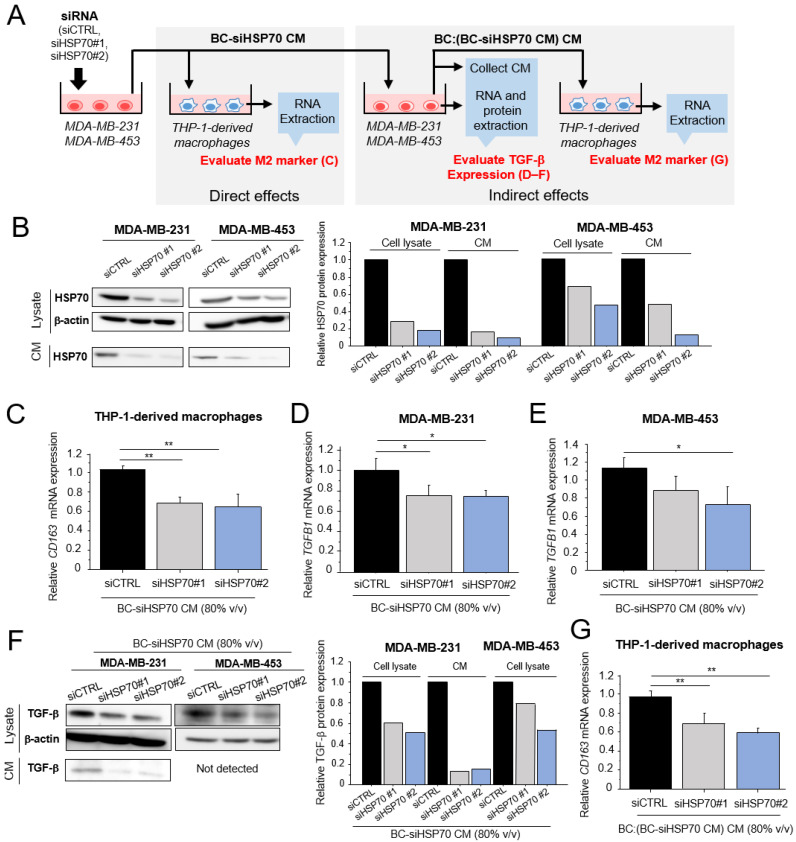
Direct or indirect effects of extracellular HSP70 from breast cancer cells on the pro-tumorigenic effects of macrophages. (**A**): Schematic image of experiments to investigate the direct or indirect effects of extracellular HSP70 on the pro-tumorigenic effects of macrophages in the present study. (**B**): HSP70 protein levels in cell lysate or CM from MDA-MB-231 and MDA-MB-453 cells transfected with HSP70-specific siRNA (siHSP70#1 and siHSP70#2). (**C**): Expression of CD163 mRNA in THP-1-derived macrophages treated with CM from MDA-MB-231 cells transfected with HSP70-specific siRNA (BC-siHSP70 CM, 80% *v*/*v*). (**D**,**E**): Expression of TGFB1 mRNA in MDA-MB-231 (**D**) and MDA-MB-453 (**E**) cells treated with BC-siHSP70 CM (80% *v*/*v*). (**F**): TGF-β protein levels in the cell lysate and CM from MDA-MB-231 and MDA-MB-453 cells treated with BC-siHSP70 CM (80% *v*/*v*). (**G**): THP-1-derived macrophages were cultured in CM from MDA-MB-231 cells treated with BC-siHSP70 CM (BC:(BC-siHSP70 CM) CM, 80% *v*/*v*), and the mRNA expression of CD163 was evaluated by real-time PCR. * *p* < 0.05, ** *p* < 0.01 vs. control (siCTRL). The data are presented as the mean ± S.D. (*n* = 3). BC: breast cancer, CM: conditioned medium. The uncropped blots are shown in Supplementary Materials.

To evaluate whether extracellular HSP70 from breast cancer cells causes macrophages to promote breast cancer progression, HSP70 knockdown was performed using siRNAs (siHSP70 #1 and #2) in MDA-MB-231 and MDA-MB-453 cells, as shown in Figure 3A. We confirmed that the HSP70 protein was successfully downregulated in both the cell lysate and the CM from MDA-MB-231 and MDA-MB-453 cells (Figure 3B).

We first investigated the direct effects of HSP70 from breast cancer cells on macrophages. When THP-1-derived macrophages were treated with CM from MDA-MB-231 cells transfected with siHSP70 (BC-siHSP70 CM), *CD163* mRNA expression was significantly decreased compared with those treated with siCTRL CM (Figure 3C). Furthermore, CM from these macrophages (THP-1:(BC-siHSP70 CM) CM, Figure S4A) slightly suppressed the cell proliferation (Figure S4B) and migration (Figure S4C) of MDA-MB-231 cells.

Next, we examined whether extracellular HSP70 from breast cancer cells enables them to secrete cytokines which can enhance the pro-tumorigenic effects of macrophages. In the present study, we focused on transforming growth factor (TGF)-β, which is known to increase the pro-tumorigenic effects of macrophages [54]. We found that the mRNA (Figure 3D,E) and protein (Figure 3F) expression of TGF-β were downregulated in MDA-MB-231 and MDA-MB-453 cells treated with BC-siHSP70 CM compared with those treated with the siCTRL CM, although we could not detect TGF-β protein in the CM from MDA-MB-453 cells. Furthermore, CM from MDA-MB-231 cells treated with siHSP70 CM (BC:(BC-siHSP70 CM) CM) significantly downregulated *CD163* mRNA expression in THP-1-derived macrophages (Figure 3G). Therefore, the pro-tumorigenic effects of macrophages are considered to be mediated by TGF-β, which is secreted from breast cancer cells in response to extracellular HSP70.

Furthermore, we suppressed the HSP70 protein on the membranes of sEVs or soluble HSP70 proteins using antibodies, in order to confirm whether HSP70 affects macrophages or breast cancer cells. When we examined *CD163* mRNA in THP-1-derived macrophages treated with CM from MDA-MB-231 cells, it was significantly suppressed in those treated with CM preincubated with the HSP70 antibody (Figure S4D). Similarly, *TGF-β* mRNA in MDA-MB-231 cells was significantly suppressed when treated with HSP70 antibodies (Figure S4E).

### 3.4. Effects of TGF-β on the Pro-Tumorigenic Effects of Macrophages

**Figure 4:**
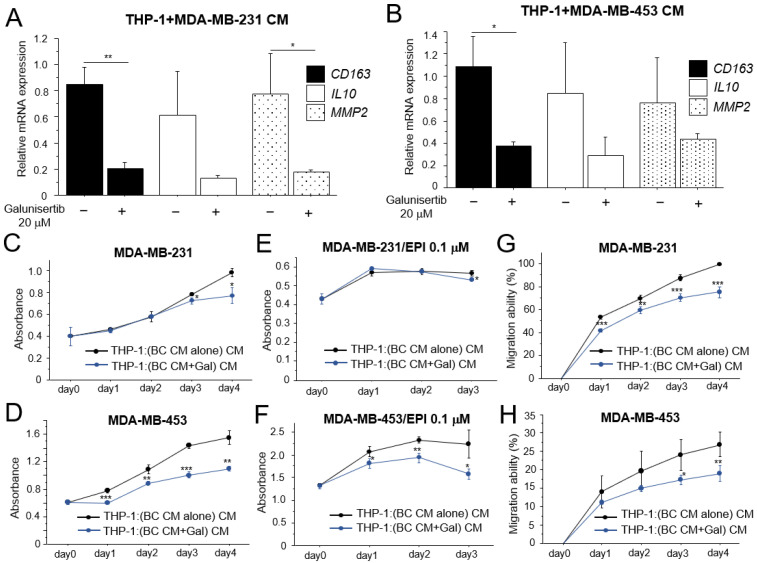
Increased pro-tumorigenic activity of macrophages by transforming growth factor (TGF)-β from breast cancer cells. (**A**,**B**): Expression of *CD163*, *IL10*, and *MMP*2 mRNA in THP-1-derived macrophages treated with CM from MDA-MB-231 (**A**) and MDA-MB-453 (**B**) cells along with Galunisertib (20 μM) (72 h). The data are presented as the mean ± S.D. (*n* = 3). (**C**–**H**): Cell proliferation (**C**,**D**), chemoresistance to EPI (**E**,**F**) and migration (**G**,**H**) assays in MDA-MB-231 (**C**,**E**,**G**) and MDA-MB-453 (**D**,**F**,**H**) cells using the CM of THP-1-derived macrophages treated with CM from MDA-MB-231 and MDA-MB-453 cells along with Galunisertib (THP-1:(BC CM + Gal) CM, 50% *v*/*v*). * *p* < 0.05, ** *p* < 0.01, *** *p* < 0.001 vs. control (free of Galunisertib (THP-1:BC CM alone)). BC: breast cancer, CM: conditioned medium, EPI: epirubicin, Gal: Galunisertib.

We next examined the significance of TGF-β in the pro-tumorigenic effects of macrophages by evaluating the expression of CD163 and interleukin-10 (IL-10) (M2 macrophage markers), as well as matrix metalloprotease 2 (MMP2), which are known to promote tumor progression [55]. When THP-1-derived macrophages were treated with CM from MDA-MB-231 (Figure 4A) and MDA-MB-453 (Figure 4B) cells in the presence of Galunisertib (Gal), an inhibitor of the TGF-β1 receptor I, the mRNA expression of these genes was significantly lower compared to those without Galunisertib (Gal). Furthermore, CM from these macrophages (THP-1: (BC CM + Gal) CM) significantly suppressed the cell proliferation (Figure 4C,D), survival in the presence of EPI (Figure 4E,F), and migration (Figure 4G,H) of MDA-MB-231 and MDA-MB-453 cells.

### 3.5. Immunolocalization of HSP70 in Human Breast Carcinoma Tissues

**Figure 5:**
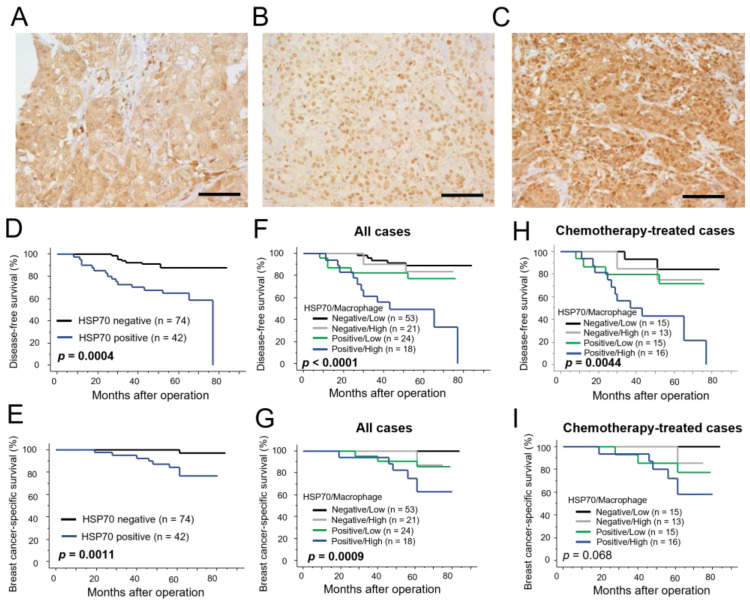
Immunohistochemistry for HSP70 in 116 breast carcinoma tissues. (**A**–**C**): Representative images of HSP70 immunostaining in the cytoplasm and nucleus of breast carcinoma cells (immunoreactivity of cytoplasm/nucleus = (**A**) positive/negative case; (**B**) negative/positive case; (**C**) positive/positive case). Bar = 100 μm. (**D**–**I**): Disease-free survival (**D**,**F**,**H**) and breast-cancer-specific survival (**E**,**G**,**I**) curves according to cytoplasmic HSP70 immunoreactivity breast carcinoma cells (**D**,**E**) or a combination of cytoplasmic HSP70 immunoreactivity and macrophage infiltration (**F**–**I**) in all patients ((**D**–**G**); *n* = 116) or in patients who had received adjuvant chemotherapy ((**H**,**I**); *n* = 59).

We then immunolocalized HSP70 in the 116 breast carcinoma tissues to investigate the clinical significance of HSP70 in breast cancer. HSP70 immunoreactivity was observed in the cytoplasm and nucleus of breast carcinoma cells (Figure 5A–C: immunoreactivity of the cytoplasm/nucleus is as follows: A, positive/negative; B, negative/positive; C, positive/positive), and we evaluated immunoreactivity in the cytoplasm of breast carcinoma cells, focusing on the activity of extracellular HSP70. In total, 36% (42 out of 116 cases) were considered positive for cytoplasmic HSP70. The correlation between HSP70 immunoreactivity and clinicopathological parameters is presented in Table 1. HSP70 immunoreactivity was significantly correlated with the stage (*p* = 0.0027), pathological T factor (*p* = 0.0029), lymph node metastasis (*p* = 0.0016), the histological grade (*p* < 0.0001), and the Ki67 labeling index (LI) (*p* = 0.0016), while it was negatively correlated with ER (*p* = 0.0060) and PR (*p* = 0.020). When we investigated cytoplasmic HSP70 expression according to intrinsic subtypes, cytoplasmic HSP70 was frequently expressed in triple-negative subtypes (*p* = 0.0050). No significant correlation was detected between macrophage infiltration and HSP70 immunoreactivity (*p* = 0.11).

Finally, we examined the correlation between cytoplasmic HSP70 immunoreactivity and clinical outcomes in breast cancer patients. Cytoplasmic HSP70 immunoreactivity was significantly correlated with an increased risk of recurrence (*p* = 0.0004, Figure 5D) and a poor prognosis (*p* = 0.0011, Figure 5E) in breast cancer patients. Furthermore, when we compared the survival curves according to HSP70 immunoreactivity and macrophage infiltration, the risks of recurrence (*p* < 0.0001, Figure 5F) and breast-cancer-specific death (*p* = 0.0009, Figure 5G) were significantly higher in patients in the cytoplasmic HSP70-positive/macrophage high-infiltration group compared with other patients. In the multivariate analysis, cytoplasmic HSP70 immunoreactivity/macrophage infiltration (*p* = 0.012), as well as the pathological T factor (*p* = 0.015) and PR (*p* = 0.043), were identified as independent prognostic factors for disease-free survival. No factors were identified as independent prognostic factors for breast-cancer-specific survival with a relative risk over 1.0 (Table 2). These tendencies were also confirmed in 59 breast carcinoma patients who had received chemotherapy. As shown in Figure 5H,I, the risk of recurrence (*p* = 0.044, Figure 5H) and-breast cancer-specific death (*p* = 0.068, Figure 5I) was higher in patients in the cytoplasmic HSP70-positive/macrophage high-infiltration group compared with other patients when the patients were limited to those who had received chemotherapy. In the multivariate analysis, cytoplasmic HSP70 immunoreactivity/macrophage infiltration (*p* = 0.011), as well as the pathological T factor (*p* = 0.0053), PR (*p* = 0.025), and HER2 (*p* = 0.026), were identified as independent prognostic factors for disease-free survival, while no factors were identified as independent prognostic factors for breast-cancer-specific survival with a relative risk over 1.0 (Table 3). Furthermore, we examined the prognostic role of macrophages according to the use of chemotherapy, and macrophage infiltration was significantly correlated with an increased risk of recurrence in patients who had received adjuvant chemotherapy (*p* = 0.027, Figure S5A); however, as we hypothesized, there was no correlation in those without chemotherapy (*p* = 0.35, Figure S5B).

## 4. Discussion

In the present study, we demonstrated for the first time that chemotherapy-induced-HSP70 release from breast cancer cells promoted the pro-tumorigenic activity of macrophages, including enhanced resistance to chemotherapy. Intratumoral macrophages are known to enhance resistance to chemotherapy in breast cancers [15,16,57], and the high infiltration of macrophages has been reported to be correlated with a low pathological complete response rate in TNBC tissues treated with neoadjuvant chemotherapy [58,59]. On the other hand, it is also important to address the altered functions of macrophages in TME following chemotherapy. In the present study, THP-1-derived macrophages cocultured with EPI-exposed breast cancer cells enhanced the proliferation, survival in the presence of EPI, and migration ability of breast cancer cells. While the differences in the chemoresistance and migration assays might be partially due to the effects of cell proliferation abilities, this finding suggests that soluble factors induced by chemotherapy might affect the surrounding macrophages and promote their pro-tumorigenic effects, causing resistance to chemotherapy. Recently, it has been demonstrated that THP-1-derived macrophages that were directly exposed to apoptotic breast cancer cells stimulated with hydrogen peroxide or cisplatin released exosomes, which enhanced tumor growth and metastasis by activating the STAT3 pathways [60]. Therefore, breast cancer cells that are damaged by chemotherapy might modify the TME by educating macrophages through soluble factors or phagocytosis, leading to the survival of cancer cells.

We next investigated chemotherapy-induced soluble factors in breast cancer cells and found that the secretion of HSP70 from breast cancer cells was promoted by the EPI, and that HSP70 was especially contained in sEVs; this finding is similar to that of a previous report using a human hepatocellular cancer cell line [61]. In addition, *CD163* mRNA expression in THP-1-derived macrophages treated with CM from breast cancer cells was significantly downregulated when HSP70 was diminished by siRNAs in breast cancer cells. This finding is in agreement with a previous study, which demonstrated that murine macrophages stimulated with bacterial HSP70 (DnaK) showed higher expression of M2 macrophage markers and promoted tumor growth in an allogeneic melanoma model [62]. However, the opposite finding has also been reported by Komarova et al. 2019, who found that THP-1-derived macrophages cocultured with human lung and colon cancer cell lines transfected with HSP70-specific shRNA showed upregulated expression of M2 markers [63]. This discrepancy may be partly due to the differences in the markers used for the evaluation of the M2 macrophages. M2 macrophages are further subdivided into the M2a, M2b, and M2c phenotypes [64] and, while Komarova et al. evaluated the expression of arginase-1, an M2a macrophage marker, we investigated the expression of CD163, which is known as a marker of M2c macrophages. In addition, CD163 is not only a cell surface marker but also serves as a functional molecule that mediates the pro-tumorigenic effects of macrophages [65]. Therefore, the expression level of CD163 in macrophages might reflect not only M2 polarization but also the enhanced function of macrophages. HSP70 might therefore upregulate CD163 expression in macrophages and induce their pro-tumorigenic effects. Furthermore, various receptors, including TLR2, TLR4, the triggering receptor expressed on myeloid cells (TREM)-1, and the receptor for advanced glycation end products (RAGE), have been reported to bind with soluble or sEVs-HSP70 and be involved in their uptake [66,67,68]. Therefore, the different receptor expression profiles in macrophages may be associated with their diverse functions in the TME. In addition, it has been reported that anticancer drugs cause the release of exosomes with HSPs from human hepatocellular carcinoma cells that induce the activation of the cytotoxic response of natural killer cells [61]. Furthermore, extracellular HSP70 has been reported to enhance resistance to chemotherapy by activating MDSC [23,28]. The effects of extracellular HSP70 on stromal cells in the tumor microenvironment are complicated, and extracellular HSP70 might have both pro-tumorigenic and anti-tumorigenic effects in the tumor microenvironment depending on the cancer type.

On the other hand, HSP70-regulated cytokines have remained largely unknown in breast cancer cells, although it has been reported that HSP70 upregulates the expression of pro-inflammatory genes via the activation of ERK1/2 and NF-κB through RAGE in human lung cancer cells [67]. In the present study, we found that breast cancer cells treated with BC-siHSP70 CM showed decreased secretion of TGF-β. In addition, decreased TGF-β in breast cancer CM resulted in suppressed *CD163* mRNA expression in THP-1-derived macrophages. TGF-β is known to polarize macrophages into an M2-like phenotype [54], and we showed that inhibition of the TGF-β receptor in THP-1-derived macrophages downregulated the mRNA expression of *CD163*, *IL10*, and *MMP2*, which were highly expressed in M2 macrophages. Importantly, the pro-tumorigenic effects of macrophages were significantly impaired when TGF-β signaling was inhibited. Considering our present findings and the previous reports, it was suggested that chemotherapy-induced HSP70 promoted TGF-β secretion from breast cancer cells, and TGF-β enhanced the pro-tumorigenic activity of macrophages, causing resistance to chemotherapy in breast cancers. Although we did not further address the detailed mechanism of TGF-β-mediated macrophage activation, TGF-β has been reported to induce the expression of urokinase-type plasminogen activator (uPA) in macrophages, which leads to the degradation of the basement membrane or the extracellular matrix by the local invasion or metastasis of breast cancer cells [69,70]. In addition, uPA promotes the release of matrix-bound basic fibroblast growth factor (bFGF) and bFGF-mediated cell proliferation and angiogenesis in breast cancer models [71,72]. Further examinations are warranted to explore the signaling pathways downstream of HSP70 and TGF-β in macrophages in breast cancer.

Finally, we investigated the significance of HSP70 in 116 breast carcinoma tissues using immunohistochemical analysis. It has been reported that circulating exosomal HSP70 levels in the blood of breast cancer patients correlate with HSP70 content within the tumor biopsies [43], suggesting that the HSP70 immunoreactivity of breast carcinoma cells possibly reflects the amount of extracellular HSP70. In addition, the present study indicated that the secretion of extracellular HSP70 and the expression of cytoplasmic HSP70 were upregulated following EPI treatment in breast cancer cells. Therefore, we hypothesized that cytoplasmic HSP70 staining of carcinoma cells partly reflected extracellular HSP70 activity [73]. Cytoplasmic HSP70 immunoreactivity was significantly correlated with the stage, pathological T factor, lymph node metastasis, histological grade, and Ki67 LI, while it was negatively correlated with ER and PR, suggesting the aggressive roles played by cytoplasmic HSP70 in breast cancer, similarly to a previous study [74]. In the prognostic analysis, cytoplasmic HSP70 immunoreactivity was significantly correlated with an increased risk of recurrence and a poor prognosis; similar findings were generated from a previous study using 191 breast carcinoma tissues of patients without lymph node metastasis [75]. On the other hand, the opposite findings have often been reported [76,77,78]; according to one study, cytoplasmic HSP70 served as a favorable prognostic factor in 60 breast cancer patients treated with anthracycline-based chemotherapy. This may be partly due to the differences in the patients’ backgrounds, the treatments performed, and the criteria for staining evaluation, and further studies using a sufficiently larger sample size with detailed reporting are needed. In particular, although the antibody used in the present study has been used in many previous studies about HSP70 [79,80,81,82,83,84], it also detects heat shock cognate (HSC) 70, an HSP70 family member, and further investigations are needed to clarify the significance of the various isoforms of HSP70 in breast cancers. Notably, when we compared the prognostic power of cytoplasmic HSP70 according to macrophage infiltration, the risk of recurrence and breast-cancer-specific death were significantly higher in the cytoplasmic HSP70-positive/high macrophage infiltration group in all patients and in patients who had received adjuvant chemotherapy, suggesting the importance of HSP70 in the pro-tumorigenic effect of macrophages in breast cancer. Macrophage infiltration was demonstrated to be a poor prognostic factor only in the patients who had received adjuvant chemotherapy. Altered TME triggered by the extracellular HSP70/macrophage axis is therefore considered to induce resistance to chemotherapy in breast cancer.

The current study’s limitations include its inability to demonstrate the detailed mechanisms of extracellular HSP70 transmission to macrophages and breast cancer cells. Using a neutralization assay, we indicated that extracellular HSP70 might affect them from the outside by binding with the receptors expressed on the cell membrane and activating signaling pathways. On the other hand, HSP70 might be partially contained inside sEVs and transported to macrophages and breast cancer cells by endocytosis. Furthermore, further experiments on the characterization of sEVs, such as those using electron microscopy and nanoparticle tracking analysis, are needed. In addition, we have not directly demonstrated that TGF-β induces the expression of M2 macrophage markers in THP-1-derived macrophages. Further experiments using purified sEVs and recombinant TGF-β might help us to better understand the detailed mechanisms of the pro-tumorigenic roles of extracellular HSP70.

## 5. Conclusions

**Figure 6:**
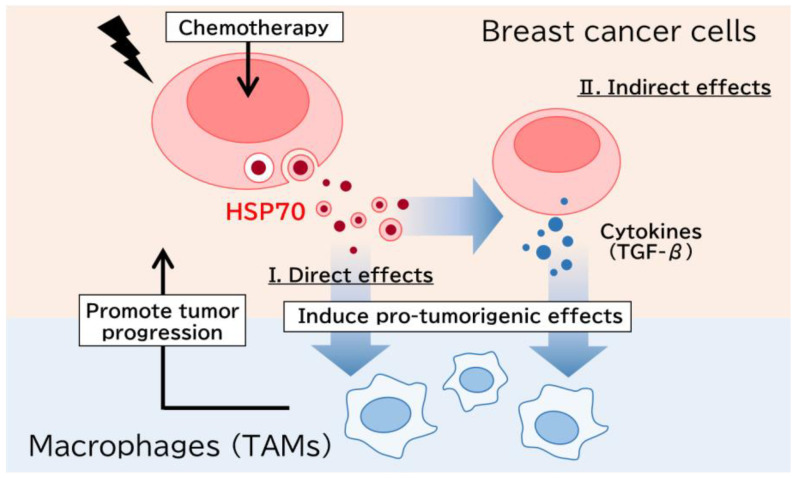
Representative image of the present study. Following chemotherapy, HSP70, which is partially contained in sEVs, is secreted from breast cancer cells. Extracellular HSP70 affects macrophages directly and regulates their polarization and pro-tumorigenic functions, while it also alters TGF-β expression in breast cancer cells and educated macrophages to promote breast cancer progression.

We demonstrated that extracellular HSP70 partially contained in sEVs affects breast cancer cells or intratumoral macrophages and causes an altered TME in breast cancer following cytotoxic chemotherapy (Figure 6). Extracellular HSP70 directly affected macrophages and regulated their polarization and pro-tumorigenic functions, while it also induced TGF-β in breast cancer cells and caused the macrophages to promote breast cancer progression. Immunohistochemical analysis demonstrated that the HSP70/macrophage axis served as a potent prognostic factor associated with resistance to chemotherapy. Targeting HSP70 may therefore be useful in regulating the TME in breast cancer tissues and improving the prognosis of breast cancer patients following chemotherapy.

## Figures and Tables

**Table 1:** Association between the immunoreactivity of HSP70 and clinicopathological parameters in 116 breast carcinomas.

		Cytoplasmic HSP70 Status		*p* Value
		Negative (*n* = 74)	Positive (*n* = 42)	
Age *		58 (29–87)	55 (27–82)	0.72
Menopause				
	Premenopause	26	15	0.95
	Postmenopause	48	27	
Stage				
	I	51	16	**0.0027**
	II	16	14	
	III	7	12	
Pathological T factor				
	pT1	57	21	**0.0029**
	pT2–4	17	21	
Lymph node metastasis				
	Negative	58	21	**0.0016**
	Positive	16	21	
Histological grade				
	1 (good)	38	7	**<0.0001**
	2 (intermediate)	28	18	
	3 (poor)	8	17	
ER				
	Negative	9	14	**0.0060**
	Positive	65	28	
PR				
	Negative	18	19	**0.020**
	Positive	56	23	
HER2				
	Negative	62	37	0.53
	Positive	12	5	
Macrophage infiltration (CD163)				
	Low	53	24	0.11
	High	21	18	
Ki67 LI * (%)		8.5 (1–53)	18 (1–60)	**0.0016**
Intrinsic subtype **				
	Luminal A	44	14	**0.0050**
	Luminal B	21	14	
	HER2 positive	5	3	
	Triple negative	4	11	

*: Data are presented as the median (minimum–max). All other values are presented as the number of cases. *p* < 0.05 is considered significant and is indicated in bold. **: The intrinsic subtype was defined according to 2011 St. Gallen surrogate definition [56]. ER: estrogen receptor, HER2: human epidermal growth factor receptor 2, LI: labeling index, PR: progesterone receptor.

**Table 2:** Summary of univariate and multivariate analyses of clinical outcomes in 116 breast carcinoma patients.

	Disease-Free Survival				Breast-Cancer-Specific Survival			
	Univariate		Multivariate		Univariate		Multivariate	
	*p* *	Relative Risk **	*p*	Relative Risk **	*p* *	Relative Risk **	*p*	Relative Risk **
Pathological T factor (pT2–4/pT1)	0.0003	**4.8** **(2.1–11)**	**0.015**	**4.3** **(1.3–14)**	**0.011**	**7.7** **(1.6–37)**	0.47	2.1 (0.28–16)
Lymph node metastasis (Positive/Negative)	**0.0028**	**3.5** **(1.5–7.8)**	0.63	1.3 (0.46–3.6)	**0.011**	**7.7** **(1.6–37)**	0.13	4.3 (0.65–28)
Histological grade (3/1, 2)	**0.0070**	**3.1** **(1.4–7.0)**	0.14	0.38 (0.11–1.4)	**0.0091**	**6.3** **(1.6–25)**	0.68	0.67 (0.10–4.5)
ER (Negative/Positive)	**0.049**	**2.3** **(1.0–5.4)**	0.20	0.46 (0.14–1.5)	*0.086*	*3.2* *(0.85–12)*	0.46	0.58 (0.14–2.5)
PR (Negative/Positive)	**0.0030**	**3.6** **(1.5–8.2)**	**0.043**	**3.1** **(1.0–9.1)**	**0.0089**	**16.0** **(2.0–128)**	*0.058*	*9.4* *(0.93–95)*
HER2 (Positive/Negative)	0.26	0.43 (0.10–1.8)	-	-	0.67	0.63 (0.079–5.1)	-	-
Ki-67 LI ***	**<0.0001**	**1.1** **(1.0–1.1)**	0.12	1.0 (0.99–1.1)	**0.0012**	**1.1** **(1.0–1.1)**	0.42	1.0 (0.97–1.1)
HSP70/Macrophage (++/others)	**<0.0001**	**5.5** **(2.5–12)**	**0.012**	**4.4** **(1.4–14)**	**0.0020**	**8.1** **(2.1–30)**	0.17	3.8 (0.55–27)

Uni- and multivariate analyses were performed using the proportional hazard model (COX). *: *p* < 0.05 (bold) and 0.05 ≤ *p* < 0.1 (italic) were considered significant and borderline significant, respectively, and were incorporated in the multivariate analysis. **: Relative risk was given with a 95% confidence interval. ***: Data were evaluated as continuous variables, and all other data were evaluated as dichotomized variables. ER: estrogen receptor, HER2: human epidermal growth factor receptor 2, LI: labeling index, PR: progesterone receptor.

**Table 3:** Summary of univariate and multivariate analyses of clinical outcomes in 59 breast carcinoma patients who had received adjuvant chemotherapy.

	Disease-Free Survival				Breast-Cancer-Specific Survival			
	Univariate		Multivariate		Univariate		Multivariate	
	*p* *	Relative Risk **	*p*	Relative Risk **	*p* *	Relative Risk **	*p*	Relative Risk **
Pathological T factor (pT2–4/pT1)	*0.066*	*2.6* *(0.94–7.2)*	**0.0053**	**5.1** **(1.6–16)**	0.22	2.7 (0.55–13)	-	-
Lymph node metastasis (Positive/Negative)	0.24	1.8 (0.68–4.7)	-	-	0.31	2.2 (0.47–11)	-	-
Histological grade (3/1, 2)	0.29	1.6 (0.66–3.9)	-	-	0.25	2.5 (0.56–9.0)	-	-
ER (Negative/Positive)	0.40	1.5 (0.60–3.6)	-	-	0.53	1.5 (0.41–5.7)	-	-
PR (Negative/Positive)	*0.059*	*2.7* *(0.96–7.4)*	**0.025**	**3.8** **(1.2–12)**	*0.077*	*6.5* *(0.81–52)*	0.19	4.3 (0.50–38)
HER2 (Positive/Negative)	*0.092*	*0.28* *(0.066–1.2)*	**0.026**	**0.18** **(0.038–0.81)**	0.35	0.37 (0.046–3.0)	-	
Ki-67 LI ***	**0.036**	**1.0** **(1.0–1.1)**	0.69	0.99 (0.96–1.0)	**0.044**	**1.0** **(1.0–1.1)**	0.41	1.0 (0.97–1.1)
HSP70/Macrophage (++/others)	**0.0012**	4.3 (1.8–11)	**0.011**	**4.8** **(1.4–16)**	**0.034**	**4.2** **(1.1–16)**	0.38	2.1 (0.41–10)

Uni- and multivariate analyses were performed using the proportional hazard model (COX). *: *p* < 0.05 (bold) and 0.05 ≤ *p* < 0.1 (italic) were considered significant and borderline significant, respectively, and were incorporated in the multivariate analysis. **: Relative risk was given with a 95% confidence interval. ***: Data were evaluated as continuous variables, and all other data were evaluated as dichotomized variables. ER: estrogen receptor, HER2: human epidermal growth factor receptor 2, LI: labeling index, PR: progesterone receptor.

## Data Availability

All data and materials presented in this article and in the supplementary documents are available from the corresponding author on reasonable request.

## Supplementary Materials

The following supporting information can be downloaded at: https://www.mdpi.com/article/10.3390/cancers15061903/s1, Figure S1: Effects of naïve breast cancer cells on the pro-tumorigenic ability of macrophages; Figure S2: Direct effects of low-dose epirubicin on the abilities of macrophages; Figure S3: Expression of HSP70 in breast cancer cells following chemotherapy; Figure S4: Direct or indirect effects of extracellular HSP70 from breast cancer cells on the pro-tumorigenic effects of macrophages; Figure S5: Association between macrophage infiltration and the clinical outcomes of 116 breast cancer patients according to the use of chemotherapy by immunohistochemical analysis; Table S1: Information on the antibody used in this study; Table S2: Sequences of the siRNA oligonucleotides and primers used in this study.
