# Supplementary material for: The Pro-Tumorigenic Role of Chemotherapy-Induced Extracellular HSP70 from Breast Cancer Cells via Intratumoral Macrophages
Source: Cancers (Basel). 2023 Mar 22;15(6):1903. doi: 10.3390/cancers15061903 (PMC10047178; doi:10.3390/cancers15061903)
Supplement: Supplementary file 1 [file cancers-15-01903-s001.zip › Supplementary Figure.pdf]

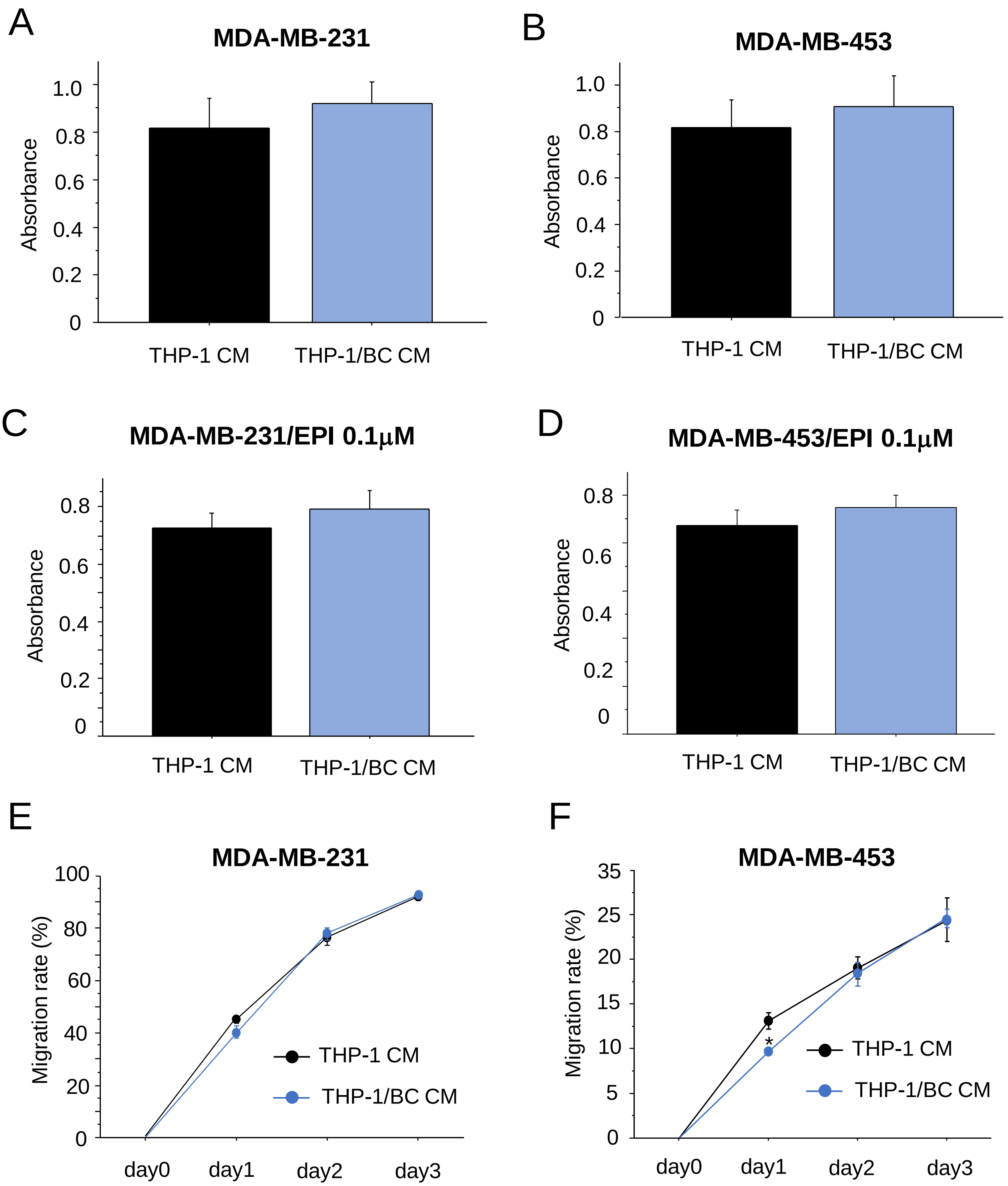

Figure S1. Effects of naïve breast cancer cells on the pro-tumorigenic ability of macrophages. (A-F): Cell proliferation (A, B), resistance to EPI (C, D) and migration (E, F) assay in MDA-MB-231 (A, C, E) and MDA-MB-453 (B, D, F) cells using CM from macrophages which had been interacted with breast cancer cells (THP-1/BC CM, 50% v/v). Cell viability was measured 72 h after CM treatment. Cell migration was examined by wound healing assay using MDA-MB-231 and MDA-MB-453 cells treated with CM (50% v/v). The data were presented as the mean ± S.D. (n = 3). *; P < 0.05 vs. control (THP-1 CM), respectively. BC; breast cancer, CM; conditioned medium.

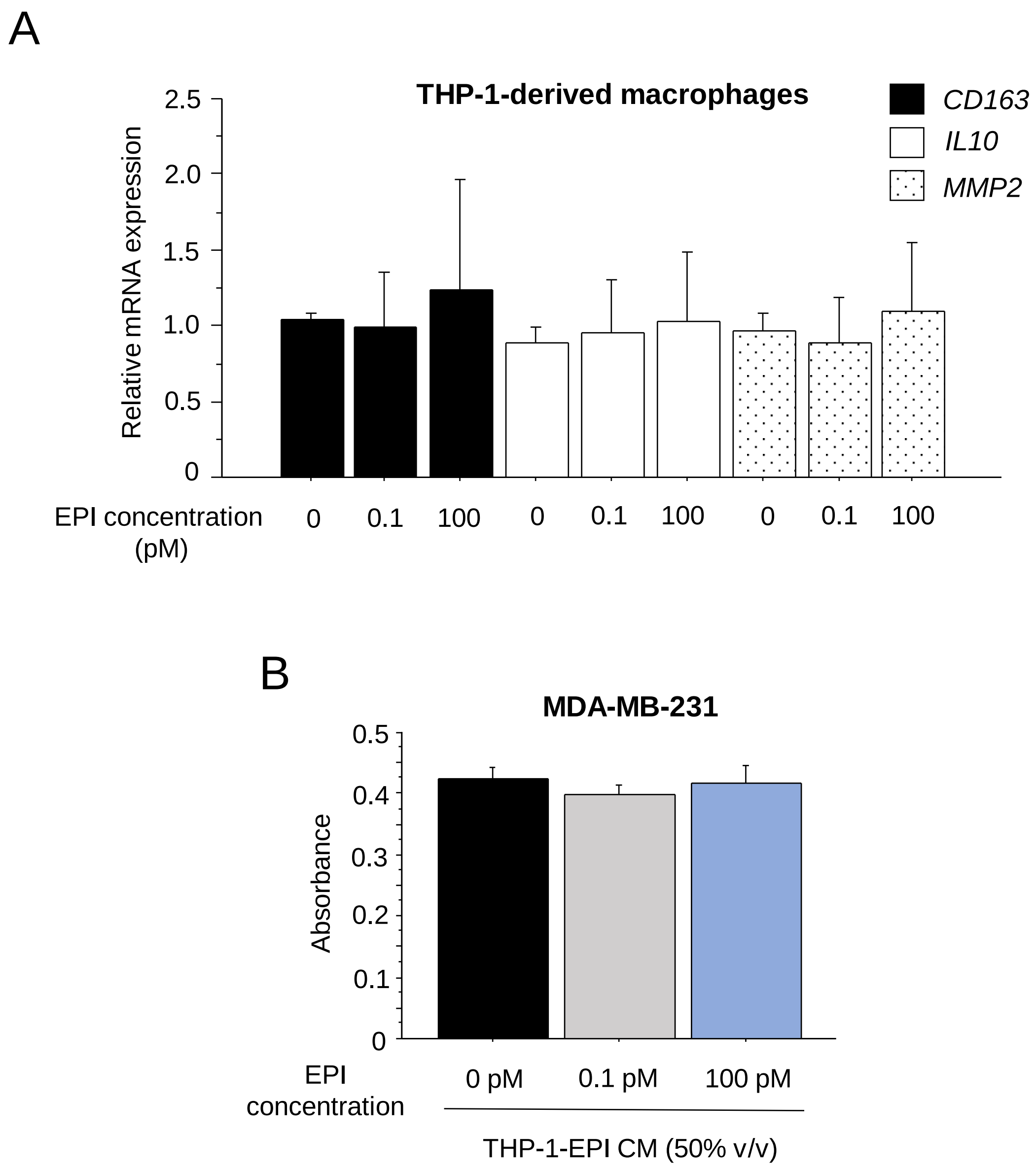

Figure S2. Direct effects of low-dose epirubicin on the abilities of macrophages.
(A): Expression of *CD163*, *IL10* and *MMP2* mRNA in THP-1-derived macrophages treated with low dose EPI (0, 0.1, 100 pM) for 72 h. (B): Cell proliferation assay in MDA-MB-231 cells using CM from macrophages treated with low dose EPI (THP-1-EPI CM, 50% v/v). The data were presented as the mean ± S.D. (n = 3). CM; conditioned medium, EPI; epirubicin.

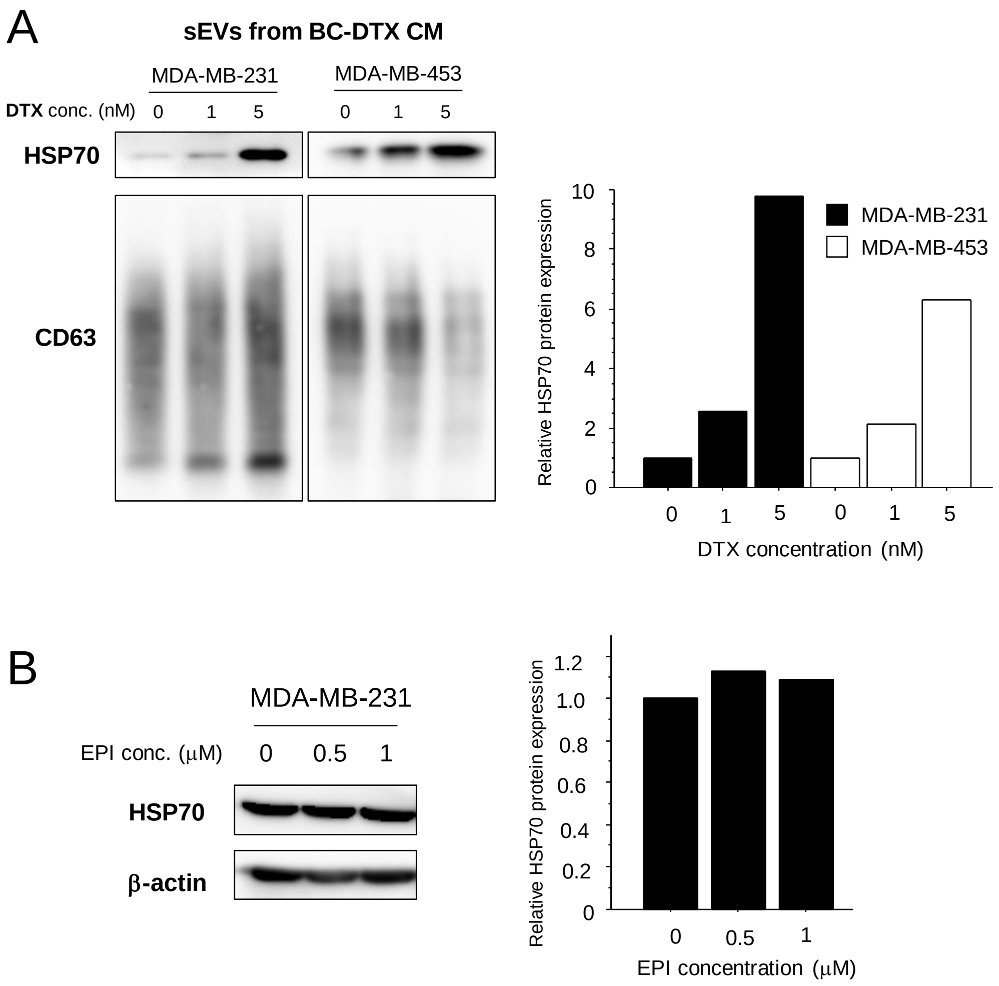

Figure S3. Expression of HSP70 in breast cancer cells following chemotherapy. (A): HSP70 protein level in sEVs from MDA-MB-231 and MDA-MB-453 cells treated with docetaxel. CD63 was used as sEVs marker. (B): HSP70 protein level in MDA-MB-231 cells treated with epiurbicin. β-actin was used as loading controls of total cell. CM; conditioned medium, DTX; docetaxel, EPI; epirubicin, sEVs; extracellular vesicles.

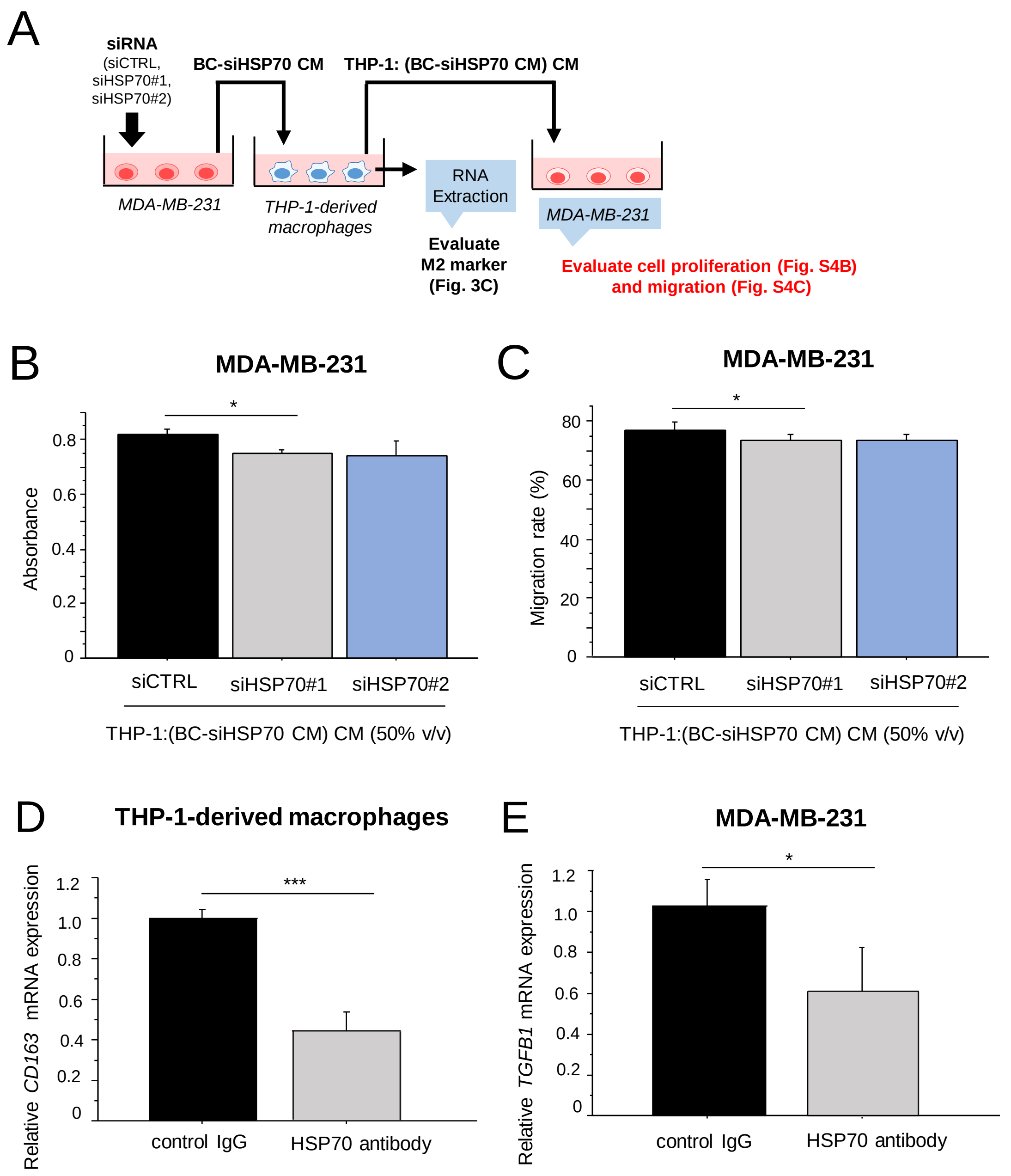

Figure S4. Direct or indirect effects of extracellular HSP70 from breast cancer cells on the pro-tumorigenic effects of macrophages. (A): Schematic image of experiments to investigate the direct effects of extracellular HSP70 on pro-tumorigenic effects of macrophages in the present study. (B, C): Cell proliferation (B) and migration (C) assay in MDA-MB-231 cells using CM from macrophages treated with BC-siHSP70 CM (THP-1:(BC-siHSP70 CM) CM, 50% v/v). (D, E): For neutralization of HSP70, CM from MDA-MB-231 cells was preincubated with HSP70 antibody or mouse IgG1 isotype control for 1h at 37℃, and THP-1-derived macrophages (D) and MDA-MB-231 (E) were cultured with CM for 24h or 48h. mRNA expression of CD163 (D) and TGF-β (E) was evaluated by real-time PCR. *; $P < 0.05$, ***; $P < 0.001$ vs. control (siCTRL or control IgG). The data were presented as the mean ± S.D. (n = 3). BC; breast cancer, CM; conditioned medium.

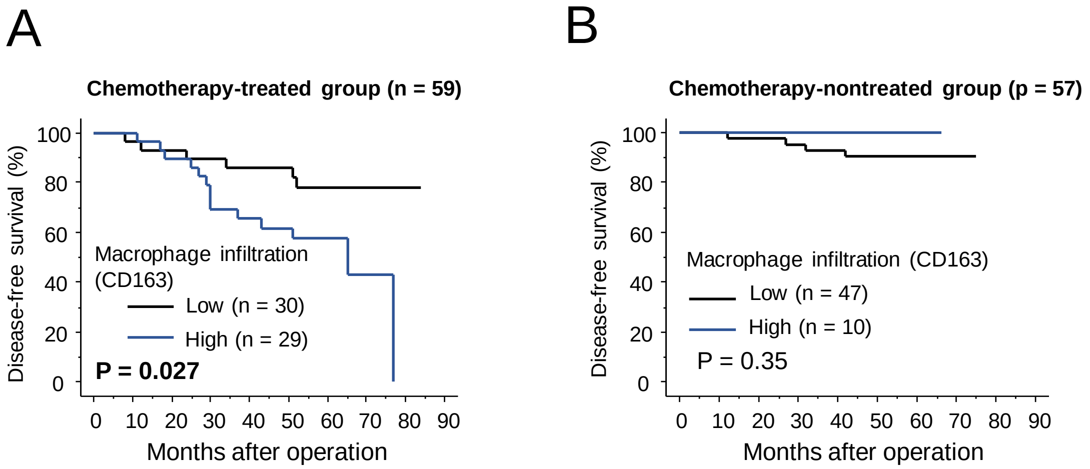

Figure S5. Association between macrophage infiltration and clinical outcomes of 116 breast cancer patients according to the use of chemotherapy by immunohistochemical analysis. Disease-free survival curve according to macrophage infiltration in breast cancer patients who had received chemotherapy (A; n = 59) or not (B; n = 57).
