# Supplementary material for: The Pro-Tumorigenic Role of Chemotherapy-Induced Extracellular HSP70 from Breast Cancer Cells via Intratumoral Macrophages
Source: Cancers (Basel). 2023 Mar 22;15(6):1903. doi: 10.3390/cancers15061903 (PMC10047178; doi:10.3390/cancers15061903)
Supplement: Supplementary file 1 [file cancers-15-01903-s001.zip › Supplementary materials-original images.pdf]

A

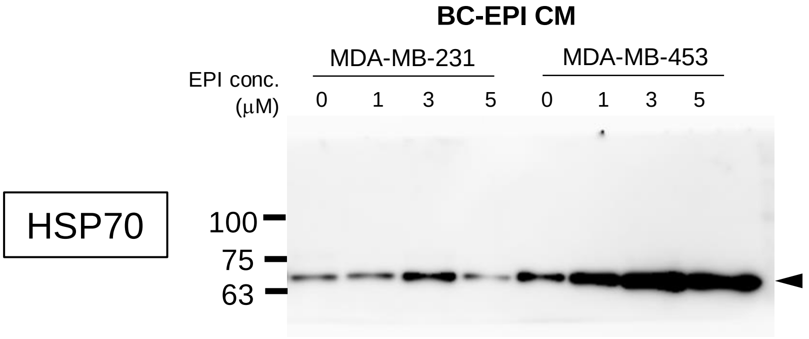

	MDA-MB-231				MDA-MB-453			
EPI conc. (μM)	0	1	3	5	0	1	3	5
Densitometry readings (HSP70)	8239.7	7309.6	16539	5265.1	15801	29097	83553	79723
Fold change	1	0.88712	2.0072	0.63899	1	1.8415	5.2879	5.0455

B

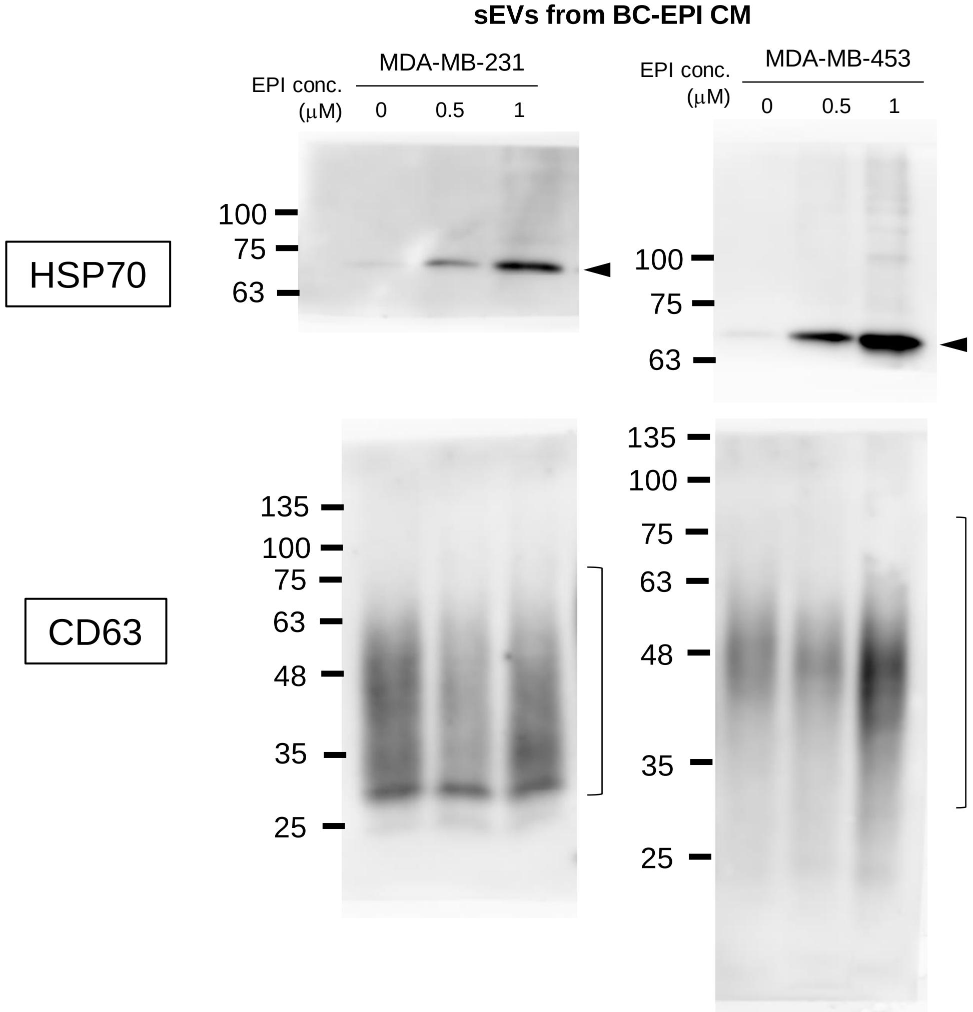

	MDA-MB-231			MDA-MB-453		
EPI conc. (μM)	0	0.5	1	0	0.5	1
Densitometry readings (HSP70)	446.51	3987.0	12143	629.16	9747.1	18862
Densitometry readings (CD63)	109817	66664	103454	39765	33136	65639
HSP70/CD63	0.004066	0.059808	0.11738	0.015822	0.29416	0.28736
Fold change	1	14.709	28.869	1	18.591	18.162

Supplemental materials; The whole blot and densitometry readings/intensity ratio of each band. The whole western blot showing all bands and molecular weight markers and densitometry readings/intensity ratio of each band shown in Figure 2A (A), B (B), Figure 3B (C), F (D), and Figure S3A (E), S3B (F).

C

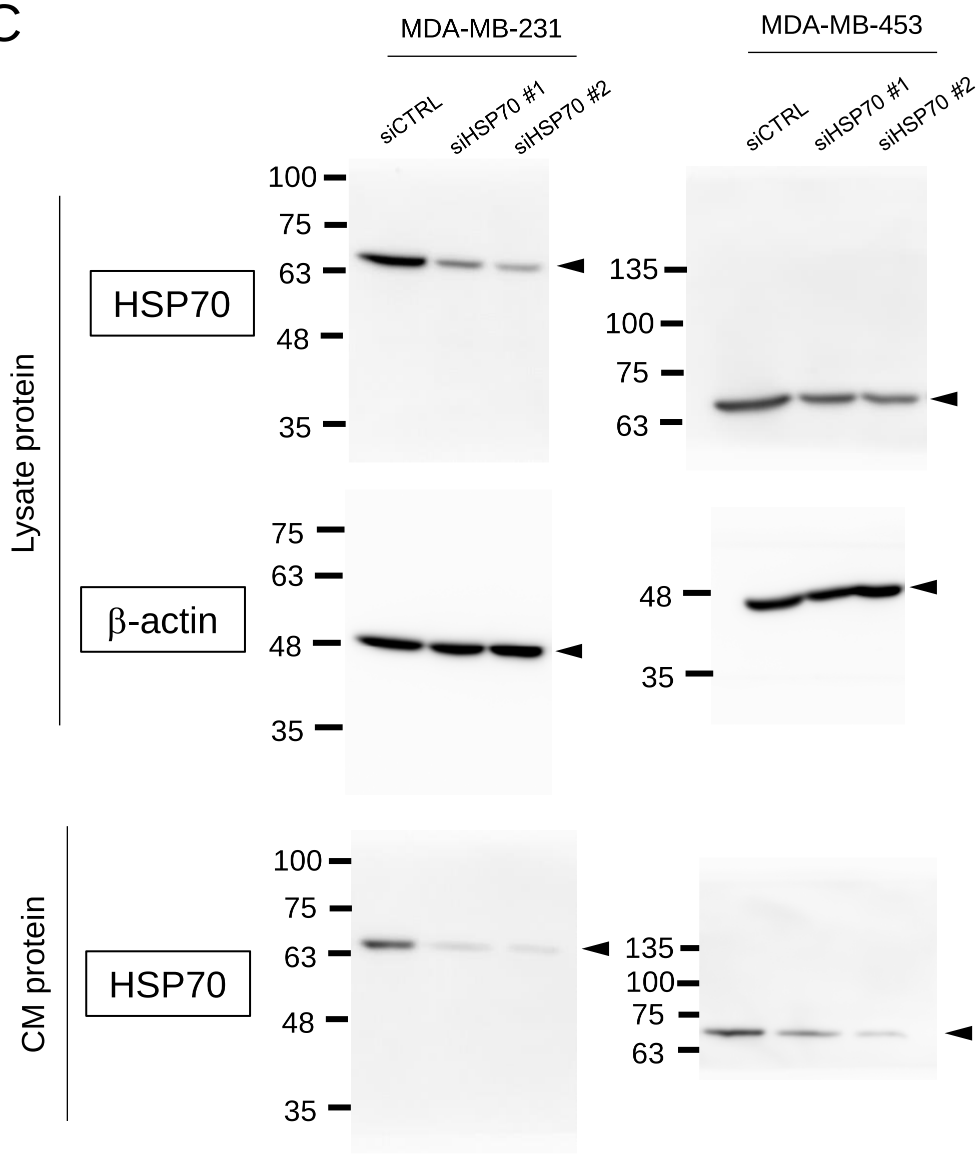

		MDA-MB-231			MDA-MB-453		
		siCTRL	siHSP70#1	siHSP70#2	siCTRL	siHSP70#1	siHSP70#2
Lysate	Densitometry readings (HSP70)	21219	6330.3	3747.0	18744	12259	9756.6
	Densitometry readings (β-actin)	18777	19506	18515	23883	22840	26540
	HSP70/β-actin	1.1301	0.32454	0.20238	0.78482	0.53671	0.36762
	Fold change	1	0.28719	0.17909	1	0.68387	0.46841
CM	Densitometry readings (HSP70)	16274	2652.4	1486.1	15064	7213.5	1906.9
	Fold change	1	0.16299	0.091322	1	0.47887	0.12659

D

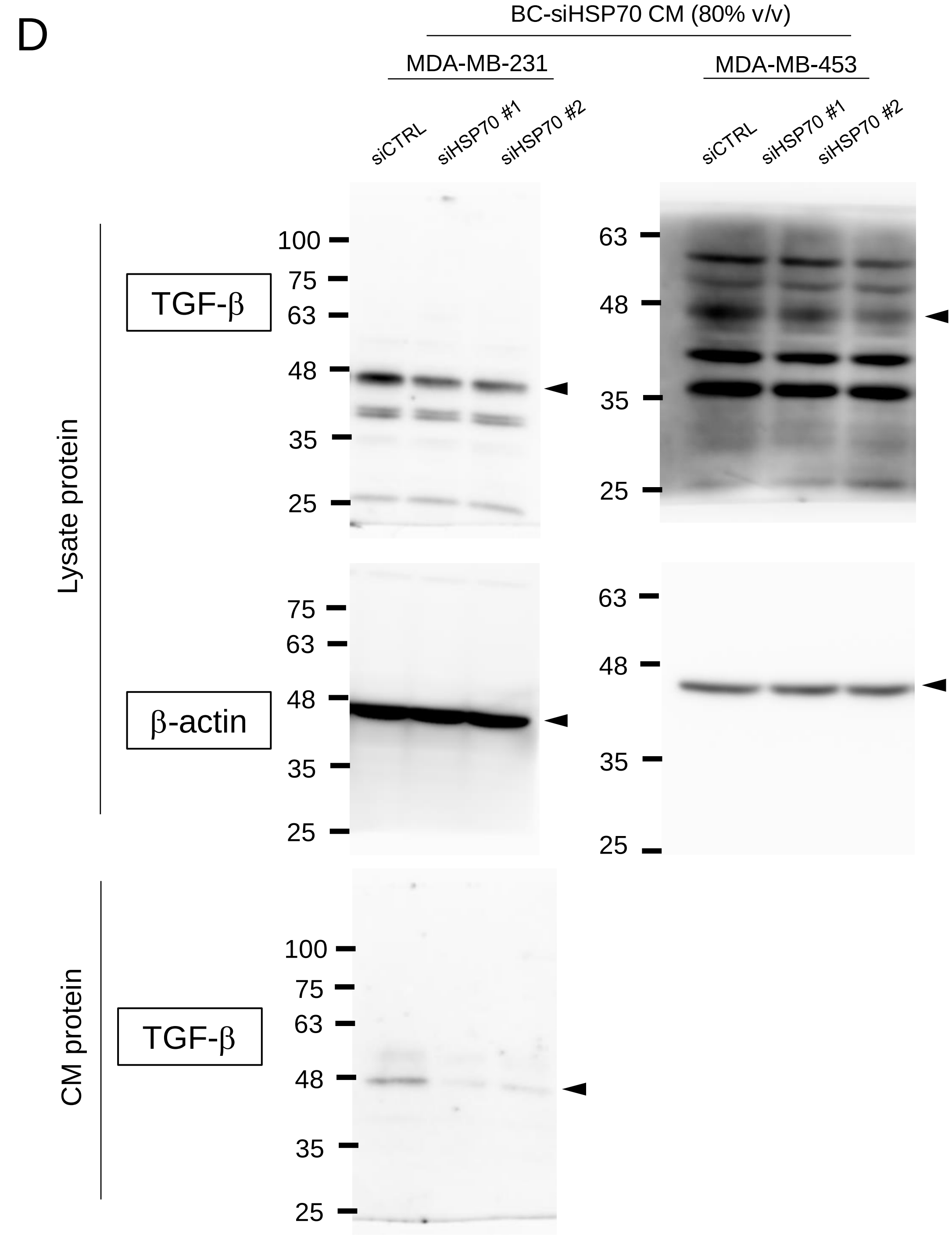

		MDA-MB-231			MDA-MB-453		
		siCTRL	siHSP70#1	siHSP70#2	siCTRL	siHSP70#1	siHSP70#2
Lysate	Densitometry readings (TGF-β)	12286	7852.8	7016.1	11345	8883.0	6122.9
	Densitometry readings (β-actin)	24245	25484	27108	19831	19708	19934
	TGF-β/β-actin	0.50674	0.30815	0.25882	0.57211	0.45074	0.30715
	Fold change	1	0.60811	0.51076	1	0.78785	0.53688
CM	Densitometry readings (TGF-β)	9496.217	1236.134	1498.669			
	Fold change	1	0.13017	0.15782			

E

sEVs from BC-DTX CM

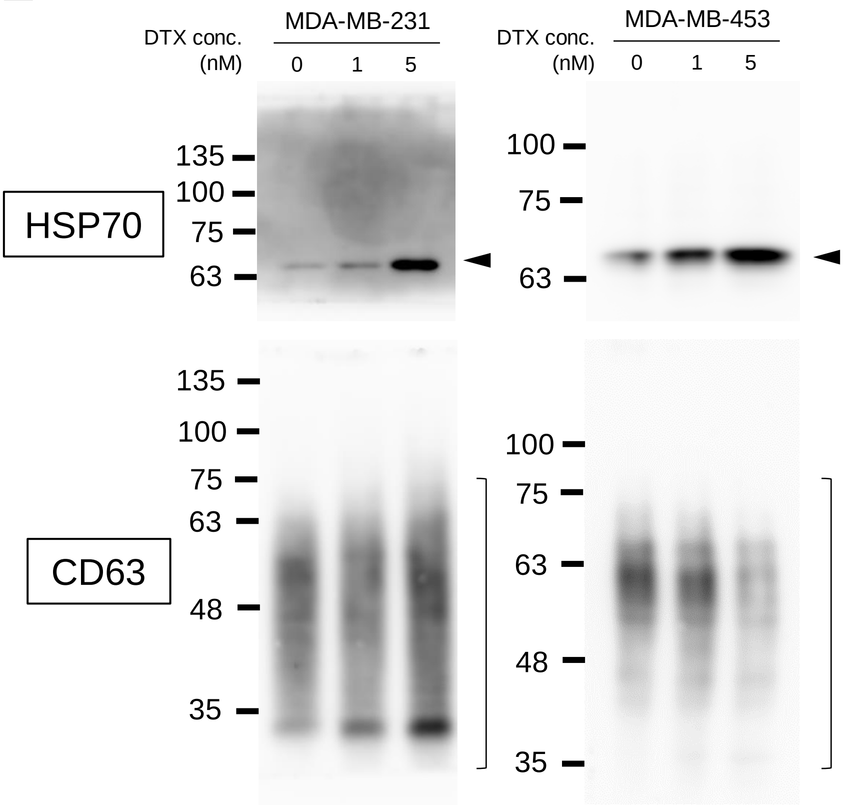

	MDA-MB-231			MDA-MB-453		
DTX conc. (nM)	0	1	5	0	1	5
Densitometry readings (HSP70)	1499.6	3569.7	18632	6808.0	13816	21856
Densitometry readings (CD63)	75375	70792	95833	71656	68864	36354
HSP70/CD63	0.019896	0.050427	0.19442	0.09501	0.20062	0.60119
Fold change	1	2.5346	9.7719	1	2.1116	6.3276

F

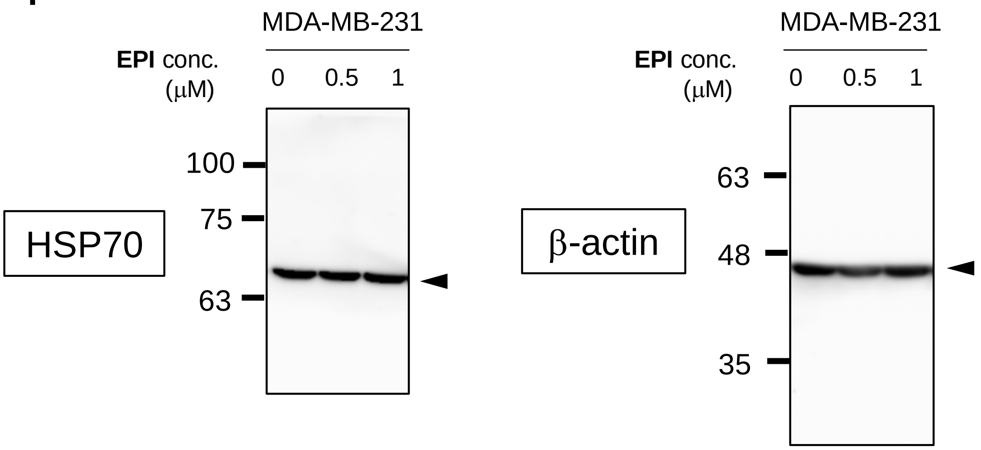

	MDA-MB-231		
EPI conc. (μM)	0	0.5	1
Densitometry readings (HSP70)	10200.761	9871.518	9995.347
Densitometry readings (β-actin)	12853.146	11000.78	11529.05
HSP70/β-actin	0.79363924	0.897347	0.86697
Fold change	1	1.130674	1.092398
