# Supplementary material for: The Pro-Tumorigenic Role of Chemotherapy-Induced Extracellular HSP70 from Breast Cancer Cells via Intratumoral Macrophages
Source: Cancers (Basel). 2023 Mar 22;15(6):1903. doi: 10.3390/cancers15061903 (PMC10047178; doi:10.3390/cancers15061903)
Supplement: Supplementary file 1 [file cancers-15-01903-s001.zip › Supplementary Table.pdf]

Supplementary Table S1. Information on the antibody used in this study.

Application	Target	Antibody***	Company	Dilution	Antigen retrieval for IHC (buffer pH)
Neutralization	HSP70	Mouse monoclonal (W27)	Santa Cruz	1:40	-
WB	HSP70	Mouse monoclonal (W27)	Santa Cruz	1:2000	-
	CD63	Mouse monoclonal (3-13)	Wako	1:4000	-
	TGF-β	Rabbit polyclonal (GTX130023)	GeneTex	1:1000	-
	β-actin	Mouse monoclonal	SIGMA-ALDRICH	1:50000	-
ICC/IF	HSP70	Mouse monoclonal (W27)	Santa Cruz	1:400	-
IHC	HSP70	Mouse monoclonal (W27)	Santa Cruz	1:400	AC pH9****
	CD163	Mouse monoclonal (NCL-L-CD163)	Leica	1:200	AC pH9****
	Ki67	Mouse monoclonal (MIB1)	DAKO	1:100	AC pH6 (Citrate buffer)
	ER*	Rabbit monoclonal (SP1)	Roche	-	NA
	PR*	Rabbit monoclonal (1E2)	Roche	-	NA
	HER2**	HercepTest -	DAKO	-	Water bath (Product component)

AC; autoclave, ER; estrogen receptor, HER2; human epidermal growth factor receptor 2, HSP70; heat shock protein 70, ICC/IF; immunocytochemistry/immunofluorescence, IHC; immunohistochemistry, NA; not applicable, PR; progesterone receptor, TGF-β; transforming growth factor beta, WB; western blotting

*; Immunohistochemistry for ER and PR was carried out using Ventana Benchmark XT system. **; Immunohistochemistry for HER2 was carried out using Herceptest (DAKO). ***; Clone number or catalog number were given for monoclonal antibody and polyclonal antibody, respectively. ****; Buffer solution was purchased from Nichirei Bio.

Supplementary Table S2. Sequences of the siRNA oligonucleotides and primers used in this study.

Target	Application	Sequences
HSPA1A, HSPA1B	siHSP70#1	Sense: 5’-CGACGGAGACAAGCCCAAG-3’<br>Antisense: 5’-CUUGGGCUUGUCUCCGUCG-3’
HSPA1A, HSPA1B	siHSP70#2	Sense: 5’-CCAUGACGAAAGACAACAA-3’<br>Antisense: 5’-UUGUUGUCUUUCGUCAUGG-3’
CD163	Real time PCR	Forward: 5’-CGGCTGCCTCCACCTCTAAGT-3’<br>Reverse: 5’-ATGAAGATGCTGGCGTGACA-3’
TGFB1	Real time PCR	Forward: 5’-AGCGACTCGCCAGAGTGGTTA-3’<br>Reverse: 5’-GCAGTGTGTTATCCCTGCTGTCA-3’
IL10	Real time PCR	Forward: 5’-ATCAAGGCGCATGTGAAC-3’<br>Reverse: 5’-CAGGGAAGAAATCGATGACAG-3’
MMP2	Real time PCR	Forward: 5’-ATAACCTGGATGCCGTCGTG-3’<br>Reverse: 5’-AGCCTAGCCAGTCGGATTTG-3’
RPL13A	Real time PCR	Forward: 5’-CCTGGAGGAGAAGAGGAAAG-3’<br>Reverse: 5’-TTGAGGACCTCTGTGTATTT-3’

HSPA1; heat shock protein family A member 1, *IL10*; interleukin 10, *MMP2*; matrix metalloproteinase-2, *RPL13A*; ribosomal protein L13A, *TGFB1*; transforming growth factor beta 1
